# Hybrid Operation Comprising Hepatic Artery Reconstruction and Endovascular Treatment for a Common-Proper Hepatic Artery Aneurysm Derived from Segmental Arterial Mediolysis

**DOI:** 10.70352/scrj.cr.25-0219

**Published:** 2025-11-15

**Authors:** Shinya Hayami, Akira Ikoma, Yoshitaka Wada, Atsushi Miyamoto, Atsushi Shimizu, Yuji Kitahata, Akihiro Takeuchi, Hideki Motobayashi, Kensuke Nakamura, Kyohei Matsumoto, Tetsuo Sonomura, Shinichi Asamura, Manabu Kawai

**Affiliations:** 1 Second Department of Surgery, School of Medicine, Wakayama Medical University, Wakayama, Wakayama, Japan; 2 Department of Radiology, School of Medicine, Wakayama Medical University, Wakayama, Wakayama, Japan; 3 Department of Plastic Surgery, School of Medicine, Wakayama Medical University, Wakayama, Wakayama, Japan

**Keywords:** common-proper hepatic artery (CHA-PHA), endovascular treatment (EVT), hepatic artery aneurysm, segmental arterial mediolysis (SAM), hybrid operation, microscopic arterial reconstruction, middle colonic artery (MCA)

## Abstract

**INTRODUCTION:**

The location and size of our patient’s hepatic artery aneurysm might suggest the possible inadequacy of endovascular treatment (EVT) only. We therefore devised a hybrid operation that included microscopic arterial reconstruction after dividing the hepatic artery with simultaneous EVT.

**CASE PRESENTATION:**

A 69-year-old man had multiple abdominal artery aneurysms that were derived from segmental arterial mediolysis. The main aneurysm, which was in the common-proper hepatic artery, was >3 cm in diameter. Complete coiling of the aneurysm might have resulted in hepatic ischemia, so we planned a simultaneous hybrid operation that comprised both hepatic artery reconstruction and EVT. First, the radiologists catheterized the common hepatic artery in preparation for aneurysm rupture. Then, hepato-biliary-pancreatic surgeons encircled and clipped 4 outflow arteries from this aneurysm: the gastroduodenal artery, right gastric artery, and the right and left hepatic arteries. Subsequently, with the help of plastic surgeons, left hepatic artery reconstruction was performed using the middle colic artery to ensure blood flow to the liver. Finally, the radiologists were able to perform coil embolization without fear of aneurysm rupture or dislocation of coils. The operation and EVT time was totaled to 567 min. The patient was discharged after 18 days without postoperative complications. Eighteen months after the operation, there has been no recurrence or regrowth of this treated aneurysm or growth of other aneurysms. Considering segmental arterial mediolysis as multiple visceral aneurysms, we performed MRA to confirm the absence of intracranial aneurysms.

**CONCLUSIONS:**

With cooperation between hepato-biliary-pancreatic surgeons, plastic surgeons, and radiologists, we performed a hybrid operation that included microscopic arterial reconstruction after simultaneously dividing both hepatic arteries and performing EVT for the hepatic artery aneurysm to avoid liver ischemia. A flexible treatment plan that draws upon a breadth of knowledge, techniques, and devices, and involves the cooperation of multiple clinical departments, is suggested to be helpful in the treatment of difficult abdominal aneurysms.

## Abbreviations

CA
: celiac axis
CHA
: common hepatic artery
CHA-PHA
: common-proper hepatic artery
EVT
: endovascular treatment
GDA
: gastroduodenal artery
LHA
: left hepatic artery
MCA
: middle colic artery
PDE
: Photo Dynamic Eye
RGA
: right gastric artery
RHA
: right hepatic artery
SA
: splenic artery
SAM
: segmental arterial mediolysis
SMA
: superior mesenteric artery
US
: ultrasound

## INTRODUCTION

Visceral artery aneurysms, including those involving CHA-PHA, are relatively rare but can be lethal if they rupture. Treatment is generally recommended in cases with an aneurysm that is ≥2 cm. EVT is less invasive, but surgical treatment is sometimes required because of the location and size of aneurysms. Maintaining organ blood flow after treating the visceral artery aneurysm is a priority, but organ ischemia, such as hepatic ischemia, may occur during EVT or surgical treatment for visceral artery aneurysms, and this might result in organ failure. We therefore considered how to maintain blood flow after treating visceral artery aneurysms as a preventive measure against organ ischemia. Here, we describe a case in which we used a hybrid operation comprising hepatic arterial reconstruction and EVT for a CHA-PHA aneurysm.

## CASE PRESENTATION

### Patient characteristics

**Fig. 1:**
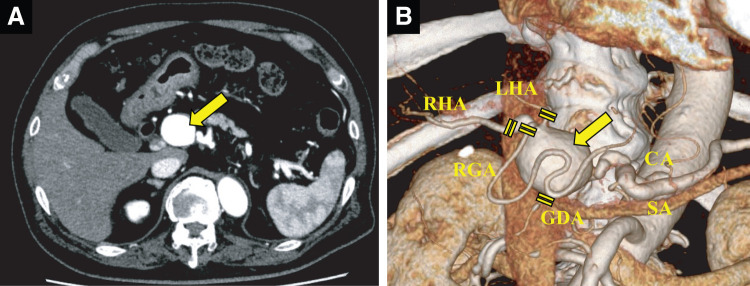
Enhanced CT showed a large aneurysm. (**A**) A large aneurysm (large yellow arrow) located at the CHA-PHA. (**B**) 3D imaging of the abdominal artery from the CA. Four outflow arteries from this aneurysm (large yellow arrow) were encircled and ligated/clipped. CA, celiac axis; CHA-PHA, common-proper hepatic artery; GDA, gastroduodenal artery; LHA, left hepatic artery; RGA, right gastric artery; RHA, right hepatic artery; SA, splenic artery

A 69-year-old man had frequent vomiting and underwent esophagogastroduodenoscopy and contrast-enhanced CT. Imaging showed multiple abdominal artery aneurysms, including those in the CHA-PHA, the root of the CA, and the branch of the RHA (**Fig. 1A**). SAM was considered to be associated with the cause of these multiple aneurysms.^1)^ The main aneurysm was ≥3 cm in diameter in the CHA-PHA. Treatment was thought to be required because of the serious risk of rupture and subsequent life-threatening intra-abdominal bleeding. This patient had a history of hypertension, which necessitated multiple antihypertensive agents (angiotensin II receptor blocker, calcium channel blocker, and antidiuretic drug), diabetes mellitus requiring an alpha-glucosidase inhibitor, and hyperuricemia requiring a selective xanthine oxidase inhibitor. The patient had a 40-year smoking habit (daily number of cigarettes: 30; Brinkman index = 1200). Laboratory data from his first visit to our outpatient clinic are shown in **Table 1**. Preoperative laboratory data indicated abnormalities related to diabetes mellitus and hyperlipidemia, but there was no evidence of liver dysfunction.

**Table 1:** Laboratory data

	Value	Unit
WBC	61.5	10^2^/μL
RBC	522	10^4^/μL
Hb	15.7	g/dl
Ht	45.3	%
PLT	23.8	10^4^/μL
APTT	28.7	sec
PT (ratio)	≥100	%
PT-INR	0.90	
TP	7.2	g/dl
Alb	4.2	g/dl
CK	52	IU/l
AST	25	IU/l
ALT	23	IU/l
LDH	157	IU/l
ALP_IFCC	81	IU/l
γ-GTP	36	IU/l
AMY	55	IU/l
Cre	0.71	mg/dl
eGFR	83.7	
BUN	10.4	mg/dl
UA	3.6	mg/dl
HbA1c (NGSP)	6.9	%
Glucose	200	mg/dl
T-cho	202	mg/dl
TG	312	mg/dl
Na	140	mEq/l
K	3.7	mEq/l
T-Bil	0.7	mg/dl
CRP	0.52	mg/dl

Alb, albumin; ALP_IFCC, alkaline phosphatase_ International Federation of Clinical Chemistry and Laboratory Medicine; ALT, alanine aminotransferase; AMY, amylase; APTT, activated partial thromboplastin time; AST, aspartate aminotransferase; BUN, blood urea nitrogen; CK, creatinine kinase; Cre, creatinine; CRP, C-reactive protein; eGFR, estimated glomerular filtration rate; Hb, hemoglobin; HbA1c, glycated hemoglobin; Ht, hematocrit; K, kalium; LDH, lactate dehydrogenase; Na, natrium; PLT, platelet; PT, prothrombin time; PT-INR, international normalized ratio of prothrombin time; RBC, red blood cell; T-bil, total bilirubin; T-cho, total cholesterol; TG, triglyceride; TP, total protein; UA, uric acid; WBC, white blood cell; γ-GTP, γ-glutamyl transpeptidase

### Preoperative conference to discuss the problems for the treatment of this CHA-PHA aneurysm

The root of the CHA appeared irregular, and in the preoperative exam, there was thought to be SAM. Wary of the possibility of intraoperative rupture of the CHA, we thought that the CHA was vulnerable during ligation, clipping, and even encircling. The most important issue in the treatment of CHA-PHA aneurysm was thought to be the potential for the incidence of liver ischemia and subsequent lethal liver failure after complete coiling of this CHA-PHA aneurysm. EVT alone might have been inadequate in this case, so a hybrid operation comprising microscopic arterial reconstruction after hepatic artery ligation/clipping and EVT was planned by consensus between hepato-biliary-pancreatic surgeons and radiologists.

The next issue to address was the selection of the feeder artery and donor hepatic artery (left or right). For the feeder artery, the MCA (2.9 mm in diameter) was the first candidate, and the SA (6 mm in diameter) was the second candidate. Meanwhile, for the donor artery, the LHA (3.2 mm in diameter) was thought to be the first candidate, and the RHA (4 mm in diameter) was the second candidate.

### EVT and surgical techniques

Radiologists first catheterized the CHA. Then, hepato-biliary-pancreatic surgeons encircled and blocked 4 outflow arteries from this aneurysm: the GDA, the RGA, the RHA, and the LHA (**Fig. 1B**). Next, assisted by plastic surgeons, LHA reconstruction was performed using the MCA or the SA to ensure hepatic blood flow. Finally, the radiologists performed coil embolization.

**Fig. 2:**
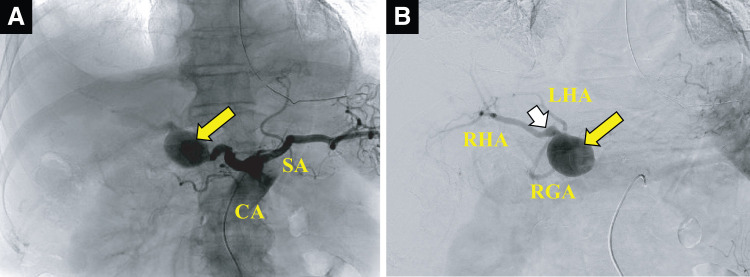
Prior to the operation, the radiologists performed angiography for catheterization of the CHA. (**A**) A large aneurysm (large yellow arrow) was located at the CHA-PHA in CA angiography. (**B**) The RHA, LHA, and RGA originated from the aneurysm. A small aneurysm was also located at a branch of RHA (white arrow). CA, celiac axis; CHA, common hepatic artery; CHA-PHA, common-proper hepatic artery; LHA, left hepatic artery; RGA, right gastric artery; RHA, Right hepatic artery; SA, splenic artery

We used a hybrid operating room that was equipped with a C-arm angiography X-ray system (Allura Clarity FD20 OR; Philips, Amsterdam, Netherlands) and an operating table (Magnus; Maquet, Rastatt, Germany). First, the CA was catheterized using a 4-Fr Rosch hepatic catheter (Medikit, Tokyo, Japan) through a 5-Fr sheath (SuperSheath; Medikit) inserted from the right femoral artery. The CA was also catheterized using a 4-Fr shepherd hook catheter (Medikit) through a 4-Fr sheath (SuperSheath; Medikit) inserted from the left femoral artery. Angiography of the CA and CHA was then performed (**Fig. 2A** and **2B**). Then, the radiologists catheterized a 2.8-Fr microballoon catheter with a 0.027-inch inner diameter (Pinnacle Blue 27; Tokai Medical Products, Aichi, Japan) via a 4-Fr Rosch hepatic catheter into the CHA to prepare for the event of an aneurysm rupture and to prevent bleeding during surgery. Care was required when catheterizing into the CA because the vessel might be fragile due to its aneurysm. Insertion of a 5-Fr balloon catheter (Selecon MP balloon catheter IIR; Terumo, Tokyo, Japan) into the CHA was difficult due to tortuous and varicose vessel dilatation and therefore it was abandoned. A 2.7-Fr microcatheter (Lantern; Penumbra, Alameda, CA, USA) was inserted into the main aneurysmal sac via a 4-Fr shepherd hook catheter (Medikit, Tokyo, Japan) in advance.

**Fig. 3:**
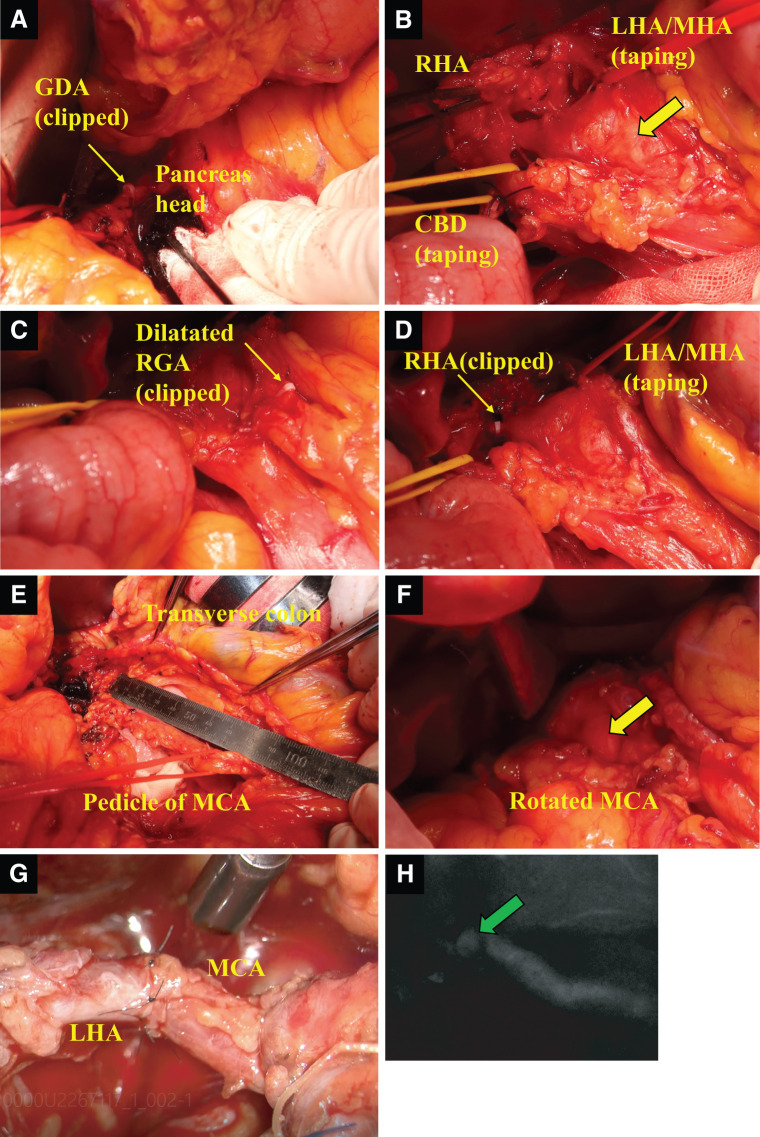
Intraoperative findings. (**A**) The GDA was clipped around the head of the pancreas (small yellow arrow). (**B**) A large aneurysm was located inside the hepatoduodenal ligament (large yellow arrow). The RHA and LHA were encircled. (**C**) The dilated RGA was also encircled and clipped (small yellow arrow). (**D**) The LHA was chosen as the distal side for arterial anastomosis, and the RHA was also clipped (small yellow arrow). (**E**) The pedicle of MCA was trimmed, and arterial anastomosis was performed between the MCA and LHA. An 8-cm MCA pedicle was finally obtained. (**F**) The obtained MCA pedicle was rotated to the cranial side without tension in the arterial reconstruction (aneurysm: large yellow arrow). (**G**) Microscopic view showing the completed arterial anastomosis between the MCA and LHA. (**H**) Good patency was confirmed by near-infrared fluorescence imaging using indocyanine green (green arrow). CBD, common bile duct; GDA, gastroduodenal artery; LHA, left hepatic artery; MCA, middle colic artery; MHA, middle hepatic artery; RGA, right gastric artery; RHA, right hepatic artery

After the radiologists finished the endovascular procedure, hepato-biliary-pancreatic surgeons performed a laparotomy by an upper-median incision. A large, beating aneurysm was detected in the hepato-duodenal ligament. The gallbladder was first resected to maintain a good surgical field, especially to identify the RHA. Four outflow arteries from this aneurysm were identified, and we encircled them in the following order: the GDA, the dilated RGA, the RHA, and the LHA (**Fig. 3A**–**3D**). Maintenance of liver perfusion was confirmed by the intraoperative Doppler US after test clamping of each of the RHA and LHA. Because intra-abdominal tissues and vessels were fragile due to SAM, ligation of these arteries, except for the LHA, was abandoned, and instead vessel clips (Hem-o-Lok; Teleflex, Research Triangle Park, NC, USA) were used, carefully avoiding injury to these arteries. For the LHA, it was decided to use arterial anastomosis because the RHA was too far to perform microscopic arterial anastomosis and its branch was also fragile due to an aneurysm.

Next, approaching the transverse mesocolon, the MCA was identified, and small vessels branching from the MCA were trimmed to make the main the trunk of MCA as long as possible (**Fig. 3E**). We finally obtained an 8-cm MCA pedicle and rotated it to the cranial side without tension in the arterial reconstruction (**Fig. 3F**). Microscopic MCA-LHA reconstruction was performed with the help of a plastic surgeon. For arterial anastomosis, we used 8-0 nylon sutures (DIADEM, polyamide monofilament suture, non-absorbable, 4 cm, double-arm; CROWNJUN, Tokyo, Japan) (**Fig. 3G**). After anastomosis, the patency of arterial reconstruction was confirmed by near-infrared fluorescence imaging (PDE-neo; Hamamatsu Photonics, Hamamatsu, Japan) using indocyanine green (Diagnogreen; Daiichi-Sankyo, Tokyo, Japan) (**Fig. 3H**).

**Fig. 4:**
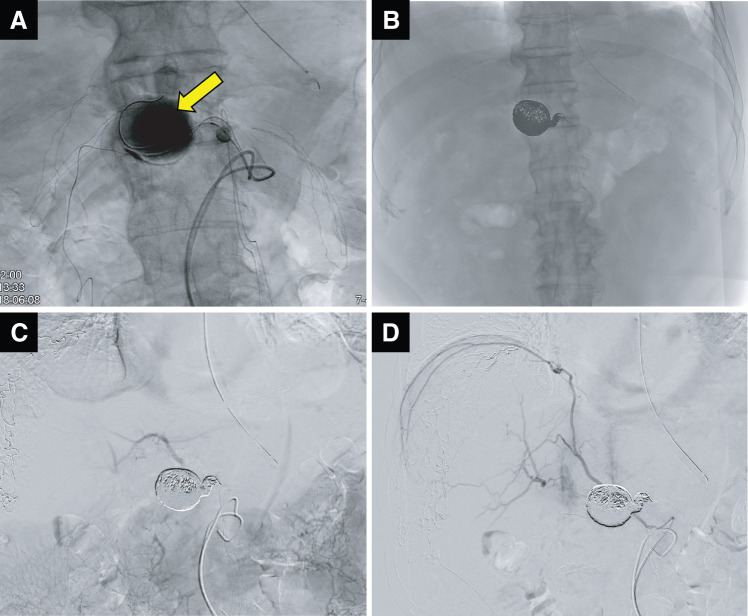
Angiography for coiling of the CHA-PHA aneurysm. (**A**) Coiling of the aneurysm (large yellow arrow). (**B**) Complete coiling of the aneurysm. (**C**) Good liver perfusion from the MCA was also confirmed by angiography of the superior mesenteric artery (SMA-MCA-LHA). (**D**) Good arterial flow of the right inferior phrenic artery was also confirmed directly from abdominal aorta. CHA-PHA, common-proper hepatic artery; LHA, left hepatic artery; MCA, middle colic artery; SMA, superior mesenteric artery

Finally, the radiologists were able to perform coil embolization without the fear of aneurysm rupture or dislocation of coils. Deflation of the balloon catheter was performed, and the aneurysm was loosely packed with 4 detachable coils (Ruby coil; Penumbra, Alameda, CA, USA), followed by coil embolization from inside the aneurysm to the beginning of the CHA with 12 detachable coils (POD packing coil; Penumbra) (**Fig. 4A** and **4B**). After the coil embolization, good liver perfusion from the MCA was also confirmed by angiography of the SMA (SMA-MCA-LHA) (**Fig. 4C**). Good arterial flow of the right inferior phrenic artery was also confirmed directly from the abdominal aorta and thought to help liver perfusion (**Fig. 4D**). There were no intraoperative harmful events such as aneurysm rupture. The operation and EVT time was totalled to 567 min. Intraoperative bleeding was 565 mL, and no red blood cell transfusion was performed.

### Postoperative management and results

**Fig. 5:**
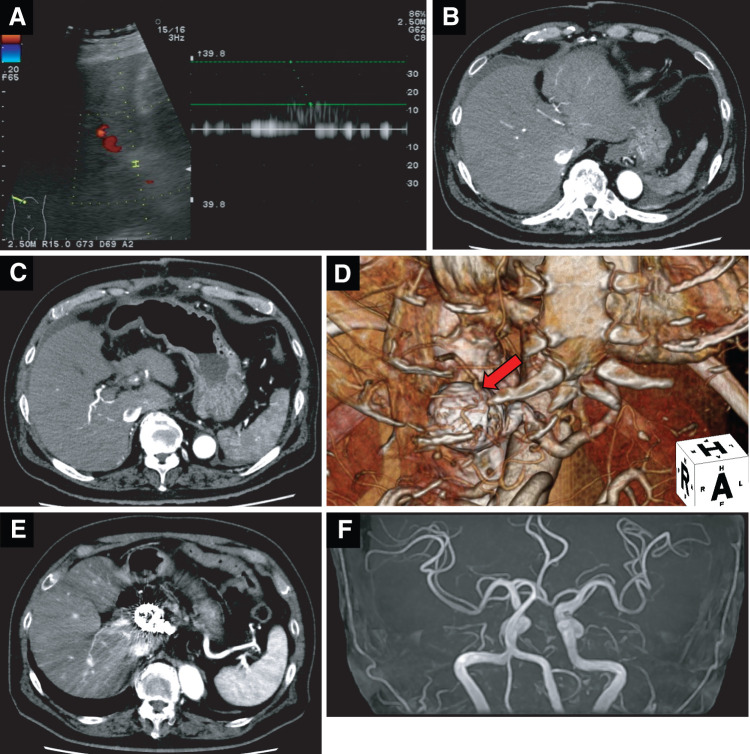
Postoperative confirmation of arterial flow, checking the regrowth of intraabdominal aneurysm, and excluding intracranial aneurysm. (**A**) Doppler US. (**B** and **C**) Enhanced CT on POD 4. (**D**) 3D reconstruction of arterial anastomosis between the MCA and LHA (red arrow: treated aneurysm). (**E**) Enhanced CT one and a half years after the operation. (**F**) MRA. LHA, left hepatic artery; MCA, middle colic artery; US, ultrasound

As an anticoagulation therapy based on our protocol after arterial reconstruction in hepato-biliary-pancreatic surgery, low-molecular-weight heparin was routinely used on PODs 1 and 2, and thereafter, unfractionated heparin was continued until POD 14. After that, no long-term antiplatelet or anticoagulant therapy was performed. To check for liver perfusion or postoperative complications such as obstruction of arterial anastomosis, daily Doppler US (**Fig. 5A**) was performed, and contrast-enhanced CT was performed on POD 4 (**Fig. 5B**–**5D**). Good patency of the LHA and adequate liver perfusion were confirmed. The patient was discharged on POD16 without surgical complications of Clavien–Dindo grade III or more. Eighteen months after the operation, there was no recurrence or regrowth of the treated aneurysm, nor growth of other aneurysms (**Fig. 5E**). Annual examinations with contrast-enhanced abdominal CT are ongoing. Considering SAM as a cause of multiple visceral aneurysms, we also performed MRA, which revealed that there was no intracranial aneurysm (**Fig. 5F**).

## DISCUSSION

We successfully performed a hybrid operation comprising microscopic arterial reconstruction after hepatic artery division and simultaneous EVT for a ≥3 cm hepatic artery aneurysm. To our knowledge, this is the first report of a hybrid treatment of this kind. The key treatment principle that we followed in this case was to maintain or normalize blood flow to the liver, originally supplied via the CHA. Recently, there have been advances in devices and techniques for EVT, and it is now generally adopted as the first-line treatment method for abdominal aneurysms.^2)^ However, in this case, the size and the location of the aneurysm meant that EVT alone might have been inadequate. EVT for hepatic aneurysms is usually performed with coils or stent grafts,^3)^ and the treatment strategy for EVT depends on whether the aneurysm is located on the proximal or peripheral side of the GDA. Complete coiling for the aneurysm at the level of the CHA is thought to be acceptable because hepatic blood flow can be maintained via the RGA and SMA–pancreatic arcade–GDA. Complete coiling of an aneurysm from the CHA to the PHA might, however, result in postoperative liver failure due to hepatic ischemia.^4)^ Revascularization would therefore be required to maintain the blood flow to the liver. To avoid hepatic ischemia, we used a microscopic arterial reconstruction technique for the hepatic artery. We have previously reported about arterial reconstruction for highly advanced hepato-biliary-pancreatic malignancies, such as biliary tract cancer or pancreatic carcinoma.^5,6)^

When planning artery reconstruction, 2 important issues require consideration: the feeder artery (proximal) and the donor artery (distal; on the liver side). Regarding the proximal artery options, based on our experience in pancreatic surgery, we considered the MCA to be the first candidate for the feeder artery because its length was thought to be sufficient for constructing an arterial anastomosis with tension. The smaller diameter of the MCA and its size mismatch (2.9 mm) with donor hepatic artery were thought to be disadvantageous in using the MCA as the feeder. However, good LHA blood flow from the MCA was confirmed by SMA angiography. If the MCA could not be used, the splenic artery was considered as the second candidate, although unfavorably, because blood flow to the stomach and pancreas could become unstable following division of the GDA and the RGA.

A second important consideration was the choice of distal artery: LHA or RHA. Based on our preoperative plan, if either the LHA or RHA could be preserved through arterial reconstruction, even after dividing both sides of the hepatic artery, the whole liver perfusion can be sufficiently secured through left and right communications in the hepatic hilum, unless the anatomical structure of hilar plate was broken.^7,8)^. If the MCA could be used for arterial reconstruction, the LHA was considered to be more acceptable than the RHA because the LHA had no aneurysm, was similar in diameter to MCA, and was near in distance.

Hybrid operations, including surgery and EVT, have been reported in the literature. Kimura et al. and Igarashi et al. respectively reported cases of multiple abdominal and renal aneurysms treated using surgery and EVT techniques; these reports were treatments against different aneurysms at the same time.^9,10)^ Jacobs et al. reported about a hybrid operation for SAM and branch aneurysms using CHA reconstruction with artificial vessels and EVT technique,^11)^ but their approach was in 2 stages rather than being simultaneous. To date, there have been no previous reports about a hybrid operation for MCA-LHA reconstruction after dividing the hepatic artery and complete coiling by EVT for a large hepatic artery aneurysm simultaneously. Our treatment procedure was thought to be unique and useful.

SAM was first reported in 1976 as a rare, non-atherosclerotic, and non-inflammatory arteriopathy.^12)^ SAM is sometimes associated with visceral and intracranial aneurysms and dissection due to pathological characteristics such as arterial medial degeneration. Vessel walls can become fragile due to SAM, so careful surgical manipulation is required. Microscopic surgery has been reported to be useful in cases of SAM.^13)^ In the present case, ligation of the CHA was judged to be difficult because the patient had SAM and there was a possibility of fragility of the CHA wall. Therefore, in addition to packing the aneurysm, we performed complete coil embolization of the CHA.

## CONCLUSIONS

We performed a hybrid operation that included microscopic arterial reconstruction after dividing both hepatic arteries and EVT for the hepatic artery aneurysm, to avoid liver ischemia. For difficult abdominal aneurysms, it is necessary to flexibly plan a treatment method using various knowledge, techniques, and devices with cooperation between multiple clinical departments.

## DECLARATIONS

### Availability of data and materials

The data that support the findings of this study are available from the corresponding author (SH) upon reasonable request.
